# Cloud Masking for Landsat 8 and MODIS Terra Over Snow‐Covered Terrain: Error Analysis and Spectral Similarity Between Snow and Cloud

**DOI:** 10.1029/2019WR024932

**Published:** 2019-07-29

**Authors:** Timbo Stillinger, Dar A. Roberts, Natalie M. Collar, Jeff Dozier

**Affiliations:** ^1^ Bren School of Environmental Science and Management University of California Santa Barbara CA USA; ^2^ Department of Geography University of California Santa Barbara CA USA; ^3^ Now at Wright Water Engineers Inc. Denver CO USA; ^4^ Now at Department of Civil and Environmental Engineering Colorado School of Mines Golden CO USA

**Keywords:** snow, remote sensing, Landsat, MODIS

## Abstract

Automated, reliable cloud masks over snow‐covered terrain would improve the retrieval of snow properties from multispectral satellite sensors. The U.S. Geological Survey and NASA chose the currently operational cloud masks based on global performance across diverse land cover types. This study assesses errors in these cloud masks over snow‐covered, midlatitude mountains. We use 26 Landsat 8 scenes with manually delineated cloud, snow, and land cover to assess the performance of two cloud masks: CFMask for the Landsat 8 OLI and the cloud mask that ships with Moderate‐Resolution Imaging Spectroradiometer (MODIS) surface reflectance products MOD09GA and MYD09GA. The overall precision and recall of CFMask over snow‐covered terrain are 0.70 and 0.86; the MOD09GA cloud mask precision and recall are 0.17 and 0.72. A plausible reason for poorer performance of cloud masks over snow lies in the potential similarity between multispectral signatures of snow and cloud pixels in three situations: (1) Snow at high elevation is bright enough in the “cirrus” bands (Landsat band 9 or MODIS band 26) to be classified as cirrus. (2) Reflectances of “dark” clouds in shortwave infrared (SWIR) bands are bracketed by snow spectra in those wavelengths. (3) Snow as part of a fractional mixture in a pixel with soils sometimes produces “bright SWIR” pixels that look like clouds. Improvement in snow‐cloud discrimination in these cases will require more information than just the spectrum of the sensor's bands or will require deployment of a spaceborne imaging spectrometer, which could discriminate between snow and cloud under conditions where a multispectral sensor might not.

## Key Points

- Some snow and cloud pixels have similar multispectral signatures, so discrimination can be difficult
- The best available cloud masks for Landsat and MODIS consistently identify snowpack boundaries as clouds
- Better snow‐cloud discrimination with multispectral sensors requires information beyond just the pixel's spectral signature

## 1 Introduction

Mapping snow cover and albedo from space is our most convenient and consistent way to observe processes that occur in the world's mountain ranges. The expanse of the mountain environment and unsettled nature of mountain weather encumber in situ and airborne data collection. Even though satellites view the mountains safely removed from these difficulties, clouds obscure parts of Earth's surface from the view of sensors in solar and infrared spectra. Knowing which pixels are snow‐covered in cloudy scenes is necessary to map the daily, weekly, and monthly changes in spatial extent, grain size, and prevalence of light‐absorbing particles of the snowpack (Dozier & Painter, 2004; Zhang et al., 2019). Accurate snow cover and albedo from remote sensing data are needed for estimating rates of snowmelt runoff (Painter et al., 2018), reconstructing the snow water equivalent (Bair et al., 2016), or understanding interannual variability and trends (Skiles et al., 2012). A serious, yet tractable, challenge to the consistent delivery of timely high‐quality information about mountain snow from spaceborne multispectral sensors is the identification of clouds in images of snow‐covered terrain. This study examines the accuracy of Landsat 8 and Moderate‐Resolution Imaging Spectroradiometer (MODIS) cloud masks over midlatitude snow‐covered mountains and highlights the difficulties for cloud masking in mountain hydrology.

Many clouds are easily identified from their visible and infrared spectra. Clouds are brighter and colder than most land surfaces except snow and ice, so for many users of remote sensing data, existing cloud masks can be trusted to identify clouds (Foga et al., 2017; Zhu et al., 2015). Since clouds tend to move, and sensors pass over the same location repeatedly, users can analyze the land surface without significant errors from cloudy pixels by using clear‐sky pixels from the clouds' neighborhood from a single scene or amalgamate clear‐sky pixels from multiple overpasses.

Most clouds are spectrally distinct from snow. The smaller sizes of the scattering droplets or ice crystals in clouds, compared to sizes of snow grains, cause the main differences between the spectral reflectance of snow and clouds (Dozier, 1989), with snow density having little effect on the snow's spectral reflectance (Bohren & Beschta, 1979; Warren, 1982). In the visible spectral region where ice and water are transparent, clouds and snow are both bright. In the shortwave‐infrared region, ice and water are moderately absorptive, so the smaller scattering elements in clouds, whether water droplets or ice crystals, usually make them brighter than snow (Warren, 2019).

Clouds frequently obstruct mountainous surfaces, and consistent cloud cover can block clear‐sky acquisitions of some areas for long periods depending on a satellite's orbit. Despite distinct radiative properties of clouds and snow, accurate, dependable cloud masks do not yet exist for use in large‐scale scientific data analysis of Earth's snow‐ and ice‐covered regions where clouds often persist. Both snow and clouds are bright, white, cold objects composed of water in either the frozen or the liquid state, and these similarities can make discrimination between snow and cloud from spaceborne multispectral sensors difficult. Persistently misclassified snow as cloud or cloud as snow affect models of snow accumulation and melt that rely on accurate snow‐covered area and albedo and local radiative conditions for energy balance modeling (Bair et al., 2016). Cloud masking algorithms perform the worst in snow‐covered mountain ranges where some clouds are identified as snow and some snow is identified as cloud (Hall & Riggs, 2007; Irish et al., 2006; Zhu & Woodcock, 2012).

Few analyses explicitly test these cloud masking algorithms for their performance over snow‐covered terrain, especially in mountains. For example, the snow and ice regions chosen to validate the Landsat 8 Cloud Cover Assessment (U.S. Geological Survey, 2016) exclude midlatitude mountainous regions and instead focus on the polar regions where validation imagery consists mostly of snow and ice with little variability in topography. Therefore, snowy mountainous regions are undersampled and binned into disparate categories when global cloud analyses create a validation data set that covers the entire globe (Foga et al., 2017; Hughes & Hayes, 2014). Mountains cover about a quarter of Earth's land surface, provide seasonal water supply for nearly two billion people, and include important ecosystems (Mankin et al., 2015; Wester et al., 2019). In North America, for example, mountains cover 24% of the area but contain ~60% of the snow (Wrzesien et al., 2018). Assessing remote sensing tools based on their global performance undervalues the importance of these mountainous regions to environmental management. Thus, the importance of mountains in supplying water and the spatiotemporal variability of seasonal snow heightens the need for better cloud masks. Since images of mountainous regions are often just partly cloudy during the snow season, usually some clear‐sky pixels exist in most scenes. These clear‐sky pixels provide information for analyses only if they can be reliably discriminated from cloudy pixels.

## 2 Methods

### 2.1 Cloud Masks Assessed

We assess the accuracy of the operational cloud masks that ship with data from the Landsat 8 Operational Land Imager (OLI; Foga et al., 2017; Roy et al., 2014) and the MODIS (Justice et al., 1998; Platnick et al., 2003). Users often start the data‐filtering step of remote sensing analyses with these products. The new Landsat standard, CFMask, now ships in the BQA file of all Landsat 8 data. The cloud mask for the MOD09GA and MYD09GA products originates from the MOD35 cloud masking code and also forms the basis for approaches that utilize additional information from time series (Friedl et al., 2002; Platnick et al., 2003). Our validation of the MODIS cloud mask utilizes data from Landsat 8 (section 2.3.1).

Snow varies at a finer spatial scale than clouds. In mountain ranges worldwide, reflectance signatures of nearly every MODIS snow‐covered pixel and 93% of Landsat snow‐covered pixels indicate the pixel includes other land covers (Selkowitz et al., 2014). In contrast, only around 15% of global cloud cover are from clouds smaller than 10 km^2^, where most of these “small” clouds are still much larger than Landsat and MODIS pixels (Wood & Field, 2011). For this analysis, we assume clouds are bigger than the sensor's pixel; hence, no fractional cloud‐covered pixels exist, whereas snow varies at a finer scale and fractional snow‐covered pixels are plentiful.

For reference, Table 1 shows the band passes and resolutions of the Landsat OLI (bands 1–9) and the “land” bands from MODIS (bands 1–7 and the cirrus band 26).

**Table 1:** Spectral Intervals (Full‐Width Half‐Maximum) and Spatial Resolution for the Landsat 8 OLI (Barsi et al., 2014; NASA/USGS Landsat 8 Team, 2014) and the MODIS “Land” Bands (NASA MODIS Team, 2003)

Band	Wavelength range (nm)	Spatial resolution (m)	Description
Landsat OLI			
1	435–451	30	ultra blue
2	452–512	30	blue
3	533–590	30	green
4	636–673	30	red
5	851–879	30	near‐infrared
6	1,567–1,651	30	shortwave‐infrared
7	2,107–2,294	30	shortwave‐infrared
8	503–676	15	panchromatic
9	1,363–1,384	30	cirrus
MODIS land bands			
1	620–670	250	red
2	841–876	250	near‐infrared
3	459–479	500	blue
4	545–565	500	green
5	1,230–1,250	500	near‐infrared
6	1,628–1,652	500	shortwave‐infrared
7	2,105–2,155	500	shortwave‐infrared
26	1,360–1,390	1,000	cirrus

### 2.2 Validation Data Sets

**Figure 1:**
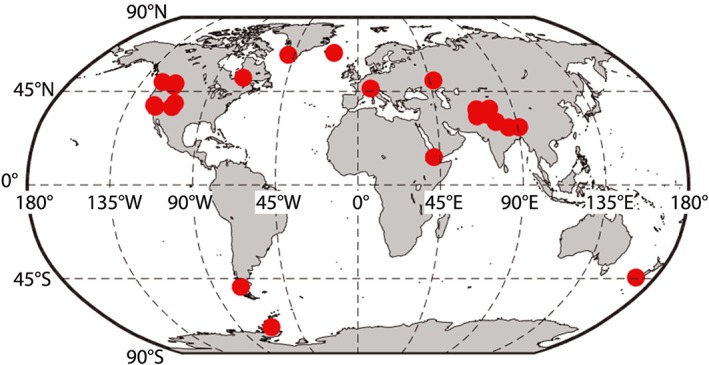
Red dots show the locations of the Landsat 8 validation images.

We used two data sets of manually classified Landsat 8 OLI data, 13 scenes that we analyzed and 13 from the U.S. Geological Survey (2016), to assess the performance of the Landsat 8 and MODIS cloud masks. Figure 1 shows the locations of the validation scenes. Landsat 8 validation data sets are a valuable assessment tool for MODIS because acquisitions from the Landsat 8 and Terra satellites occur within 20 min of each other on similar near‐polar descending orbits. Manual classification of pixels is more difficult on the coarser 250‐ to 1,000‐m resolution MODIS data compared to the 30‐m OLI data. The close acquisition times of the two satellites minimizes misinterpretation caused by cloud movement.

Any manual classification approach that relies on Landsat imagery for creating validation data will misclassify some pixels. Foga et al. (2017) estimate the difference between individual analysts at 7%. Thin clouds that do not have distinguishing reflectance in OLI band 9 and some small clouds fewer than ~10 pixels are difficult for a human to classify. Snow pixels with fractional snow‐covered area below 50% can be difficult to identify. Along the borders of a snowpack, some of the pixels with lower snow‐covered area are mistakenly excluded. Some snow‐free pixels may mistakenly be included because exactly tracing the boundary of every snowpack and cloud in 30‐m spatial data is impossible.

Within the 13 scenes that we selected, we manually masked snow, cloud, and snow‐free pixels. Using U.S. Geological Survey (USGS) Earth Explorer (https://earthexplorer.usgs.gov/), we previewed false‐color scenes over the western United States and the Himalaya to find images that included a variety of cloud types and areas of cloud cover over snow, forest, and bare ground. Scenes with complete cloud cover or no clouds were excluded. Within chosen scenes, we randomly located regions that contained cirrus clouds, thin clouds, and opaque clouds colocated with snow‐covered mountains. Pixels were then manually classified as cloud, snow, or “neither,” that is, clear‐sky and snow‐free, containing either land or open water. Reference layers were created in ArcGIS using various techniques to enable visualization of each cover type. Snow pixels were identified using natural color images of Landsat 8 OLI bands 4, 3, and 2, false color images of bands 6, 5, and 4, and a mask of the Normalized Difference Snow Index (NDSI; Dozier, 1989) from bands 3 and 6. Opaque clouds were identified using the same natural color bands 4, 3, and 2 along with grayscale images of bands 2, 4, and 5 and false color images of bands 2, 4, and 5. Images from the cirrus band (band 9) were used to identify clouds high enough in the atmosphere where minimal water vapor existed above them. Pixels that were manually identified as more than one cover type were excluded from the reference masks.

The second validation data set of 13 scenes came from the 2014 USGS SPARCS manual classification data set (U.S. Geological Survey, 2016) of Landsat 8 OLI subscenes of 1,000 × 1,000 pixels, with each pixel identified as snow, cloud, cloud shadow, water, land, or bad data. Of 80 scenes in this data set, 13 included significant snow within the manual masks. We used these 13 scenes and the 13 that we classified to assess the performance of the operational CFMask in areas of snow cover. We categorized water and land pixels as neither given our focus on the ability to distinguish clouds from snow. We excluded the pixels labeled as cloud shadows from the analysis because that class aggregates both shaded snow and shaded vegetation and soil.

Both sets of validation data were generated on Pre‐Collection Landsat data that were then aligned to Collection 1 Landsat scenes. The manually identified pixels were compared to the pixel classifications in the BQA file of the Collection 1 data (U.S. Geological Survey, 2017), specifically the CFMask bits for cloud and cirrus, which were merged into a single binary cloud mask. Most users will employ this default CFMask, which Foga et al. (2017) determined to be the best cloud mask for Landsat 8 data.

### 2.3 Error Assessment Metrics

We statistically evaluated the performance of the extant Landsat OLI snow and cloud algorithms and MODIS cloud algorithm against the manual masks from OLI. We compared the algorithms' classifications for each class of interest (snow, cloud, and neither) for each pixel to the manual classification. For each class of interest (snow, cloud, and neither), we categorized each pixel as follows:

1. *True positive—TP*: Both the automated mask and analysts classify the pixel as the class of interest.
2. *True negative—TN*: Both the automated mask and analysts classify the pixel as not the class of interest. The classification of the analyst and the automated mask do not have to match.
3. *False negative—FN*: Analysts classify the pixel as the class of interest, and the automated mask classifies it differently.
4. *False positive—FP*: Automated mask classifies a pixel as the class of interest, and the analysts classify it differently.

For each scene and for all scenes aggregated together, the total counts of each category (TP, FP, TN, and FP) for each class of interest (snow, cloud, and neither) are totaled. The following metrics are used in the error assessment of the algorithms (Olson & Delen, 2008):

(1) $$\text{Precision}=\frac{\mathrm{TP}}{\mathrm{TP}+\mathrm{FP}}$$

(2) $$\text{Recall}=\frac{\mathrm{TP}}{\mathrm{TP}+\mathrm{FN}}$$

(3) $$F=2\frac{\text{Precision}\times \text{Recall}}{\text{Precision}+\text{Recall}}=\frac{2\mathrm{TP}}{2\mathrm{TP}+\mathrm{FP}+\mathrm{FN}}$$

*Precision* represents the probability that a pixel identified as a class is indeed that class. *Recall* represents the probability of detection of a pixel of the class of interest. The *F* score balances the two metrics. Some error analyses use *Omission* (missing the class of interest) and *Commission* (misclassifying the pixel as the class of interest) instead. Their correspondences are as follows: *Omission* = 1 − *Recall* and *Commission* = 1 − *Precision*. We use Precision and Recall because they more directly convey the ability of the product to mask scenes. A number closer to 1.0 corresponds to better performance.

#### 2.3.1 Using Landsat 8 Data for MOD09GA Error Assessment

To create a validation data set directly with MODIS data, the 250‐ to 1,000‐m spatial resolutions of MODIS land bands (Table 1) are too coarse for consistent, accurate manual validation. The boundaries of clouds and snowpacks are hard to identify in the same contextual way a human analyst can with the spatial and contextual information in a Landsat scene with 30‐m spatial resolution. The similar overpass times of MODIS Terra and Landsat 8 enable the use of Landsat 8 validation data to assess the MOD09GA cloud mask. While the clouds are not exactly in the same places in the two acquisitions, the large MODIS pixel size buffers against cloud movement, and the typical 20‐min gap between acquisitions means most clouds are nearly in the same locations. Only MODIS pixels that are fully covered by pixels from the Landsat reference mask are used. After alignment and removal of partially covered MODIS pixels, we were left with 21,023 manually classified MODIS pixels. The Landsat reference mask is ternary, with each pixel either *cloud*, *snow*, or *neither*. In coarsening the Landsat mask to MODIS pixel size, the resulting pixels have fractional covers of each category. We therefore classified the MODIS pixel as cloud or snow‐covered if it had fractional coverage of 50% or more of Landsat reference mask pixels of that class. This coarsening resulted in 2,839 snow, 1,881 cloud, and 16,249 neither MODIS reference mask pixels. The MOD09GA cloud mask flags pixels as *clear*, *cloudy*, *mixed*, or *not sure*. We combined the MOD09GA classifications: pixels flagged as cloudy or mixed as cloud‐covered and those flagged as not sure or clear as clear. The “not sure” pixels comprised 3.6% of the data set.

#### 2.3.2 Analyses of Reflectance Spectra

A plausible explanation for the difficulty that algorithms have in masking clouds over snow‐covered terrain is that some clouds and some snow are spectrally too alike to discriminate, specifically for algorithms that make classification decisions from spectral thresholding and index tests. In our analysis, we highlight the difficulty in separating clouds and snow by extracting all snow and cloud spectra from the Landsat 8 validation data and exploring the similarities and differences between the snow and cloud classes.

To create these snow and cloud spectral libraries for analysis, we used the data set of manually masked Landsat scenes to extract top‐of‐atmosphere reflectance spectra of the snow and cloud pixels from all the scenes. This extraction resulted in spectral libraries of 4.6 million snow pixels and 3.3 million cloud pixels.

To test the hypothesis that most snow and cloud pixels are spectrally distinct, but some are similar, we performed *k*‐means unsupervised classifications of equally sized random subsets of the snow and cloud pixels from the spectral library. *k*‐means classification (Hastie et al., 2009) maximizes similarity within each cluster while maximizing dissimilarity between clusters. Using *k*‐means in this manner gives an overview of similarity between known classes of pixels. By running the same classification on different random subsets of pixels, we highlight the similarity or dissimilarity in spectral signatures between the classes. Because equally sized subsamples of pixels from the two spectral libraries are used, two classes of pixels that are inseparable and spectrally identical would lead to two clusters output from the classifier that are a 50/50 mix of both class types. Conversely, two classes of pixels that are spectrally unique will be output from the classifier as two clusters that each have 100% membership from one of the input classes. How various runs compare between these two extremes enables us to determine what may lead to the similarity or dissimilarity between classes.

Snow and cloud pixels were classified with *k*‐means using the spectral angle as the distance metric (Kruse et al., 1993) and the number of output clusters set to two. Pixels are clustered based on their similarity in angle away from the origin in multidimensional space, where each Landsat 8 band reflectance is a dimension. Given two spectral vectors $\vec{R_{1}}$ and $\vec{R_{2}}$, the cosine of the spectral angle ∠_*λ*_ is the dot product of the two vectors divided by the product of their Euclidean norms:

(4) $${∠}_{\lambda }=\cos^{-1}\left(\frac{\vec{R_{1}}\cdot \vec{R_{2}}}{{\left\|\vec{R_{1}}\right\|}_{2}\times {\left\|\vec{R_{2}}\right\|}_{2}}\right)$$

Two pixels of the same class with reflectances of different magnitudes but similar spectral shape have similar spectral angles.

The magnitudes of the reflectances may be as important as the spectral angle for the most similar snow and cloud pixels. When detecting clouds or snow in Landsat 8 OLI bands 6 and 7, ice or water absorption dominates the signal with similar spectral shape, yet reflectance magnitude is sensitive to the size of the droplet or crystal. To test if the absolute magnitude of the signal is also important for snow and clouds, a second run of *k*‐means was performed using the Euclidean norm of the differences, ${\left\|\vec{R_{1}}-\vec{R_{2}}\right\|}_{2}$, as the metric for clustering and the number of output clusters again set to two. Landsat OLI bands 1–7 were used for these classifications. Band 9 was omitted because the top‐of‐atmosphere cirrus band reflectance depends on the atmospheric water vapor above each pixel; band 9 misidentifies many high‐elevation snow‐covered pixels as cirrus.

We performed three runs of *k*‐means classification: all snow and cloud pixels, snow and similar cloud, and the snow and dissimilar cloud. “Similar” cloud pixels have SWIR reflectances (bands 6 and 7) that are less than or equal to the 99th percentile of snow reflectances in the same bands from the snow pixel library. Thirty‐five percent of the cloud spectral library met these criteria and were identified as similar cloud and snow. We used the 65% of the cloud spectral library that were excluded from this data set as the data for the unsupervised classification of dissimilar cloud and snow. Results from the unsupervised classification were compared with Precision, Recall, *Overall Accuracy*, and *Separation*, where the cluster is classed as the majority fraction of reference pixels it contains. Precision and Recall are the same tests used to assess the errors in the snow and cloud masks; Separation is the distance between the centroids of the two clusters using the same distance metric that the classification was run on. Because we have restricted the classification to two classes of the same size, Overall Accuracy (*TP*+*TN*)/(*TP*+*TN*+*FP*+*FN*) is also a useful measure.

### 2.4 Modeling Reflectance of Snow and Cloud Pixels

We model the spectral signatures of water clouds, ice clouds, and mixed clouds based on the refractive indices of ice and water, the sizes of the water droplets and ice crystals, the clouds' water equivalent, wetness in the case of mixed clouds, the illumination geometry, and the reflectance of the surface beneath the clouds. We model the spectral signature of snow based on effective optical radius, dust size and concentration, the fractional cover of the snow in the pixel, and the reflectance of the surface comprising the remainder of the pixel. For each pixel analyzed, we invert the models to estimate cloud properties and snow properties that match the measured reflectances best. The physically based scattering model (Warren & Wiscombe, 1980; Wiscombe & Warren, 1980) is forced by the Mie scattering parameters for a sphere (Bohren & Huffman, 2007; Nussenzveig & Wiscombe, 1980) and the complex refractive indices of ice (Warren & Brandt, 2008) and water (Hale & Querry, 1973; Kou et al., 1993) to generate snow and cloud spectra with spherically equivalent radii that encompass the size distributions in clouds and snow. The Simple Model of the Atmospheric Radiative Transfer of Sunshine (Gueymard, 2005) generates the irradiance spectra illuminating the modeled snowpack along with atmospheric transmittance and thereby top‐of‐atmosphere reflectance. The snow spectra are linearly mixed with soil and vegetation spectra from the ECOsystem Spaceborne Thermal Radiometer Experiment on Space Station (ECOSTRESS) spectral library (Baldridge et al., 2009; Meerdink et al., 2019) to compute the reflectance of partially snow‐covered pixels. The modeled clouds are not opaque, and the same spectral library is used to set the background reflectance underneath clouds.

We test the hypothesis that some snow and cloud pixels can have spectra from Landsat 8 OLI that match pixels of the opposite class. Snow pixels that CFMask classes as clouds and cloud pixels that CFMask classes as snow are considered in inversion models to solve for the best matches of cloud and snow properties that match the measurements. The inverted properties are then used to model the reflectances throughout solar spectrum at the wavelengths of the AVIRIS‐NG airborne instrument (JPL AVIRIS Team, 2018), 380 to 2,500 nm at 5‐ to 6‐nm resolution, to investigate if measurements from a spectrometer would distinguish snow from clouds, whose discrimination might be missed by Landsat 8 OLI.

## 3 Results

### 3.1 Assessment of Errors in CFMask and MOD09GA Cloud Mask

**Figure 2:**
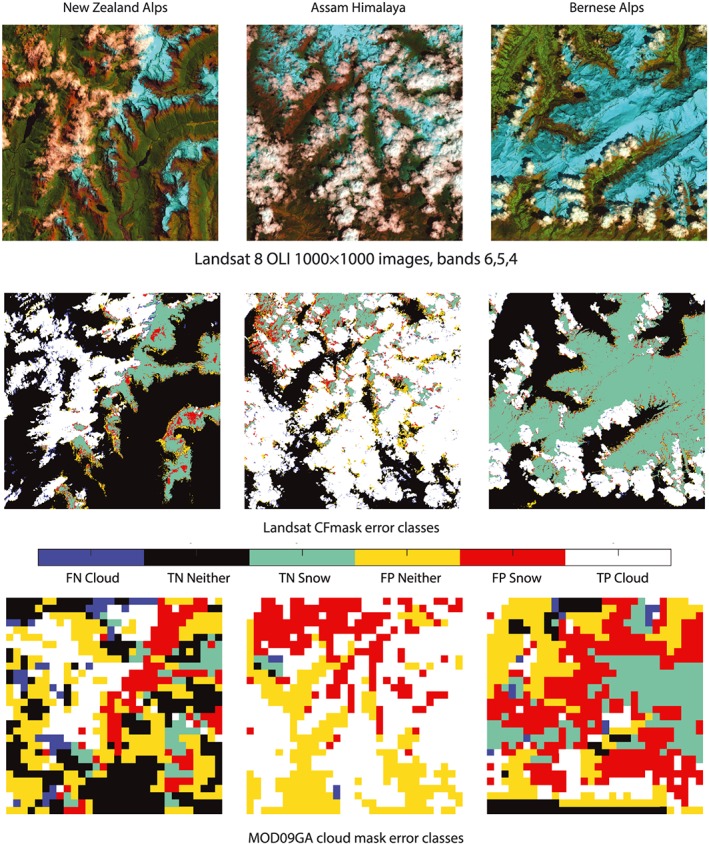
Examples of CFMask and MOD09GA cloud mask performance over three areas with varying degrees of cloud cover. Pixels are colored by surface type and identified as false negative (FN), true negative (TN), false positive (FP), or true positive (TP) in the cloud mask. (left column) New Zealand Alps: Climax Peak (2,446 m) in the Pyke and Dart River Basins, clouds and snow about equal. (middle column) Assam Himalaya: below Chura Kang (6,300 m) in Bhutan, cloud cover extensive but snow visible. (right column) Bernese Alps: Lötschental and Bietschhorn (3,924 m), mostly snow with few clouds.

Figure 2 shows three examples of our approach for analyzing cloud masks. Supporting information Table S1 provides error statistics for all 26 scenes, along with each acquisition's terrain characterized by the percent cover of mountain landforms from the global Hammond landform regions (Karagulle et al., 2017), which quantifies Earth's mountainous locations at 250‐m horizontal scale. Two of the USGS SPARCS masks with snow have no mountain cover, and their inclusion expands the range of snow spectra analyzed. One was an acquisition at high illumination angle from the Greenland ice sheet, and the second was of cropland with variable snow‐covered area. A USGS SPARCS mask of the Antarctic Peninsula Cordillera is outside the boundary of the Hammond data set, so no mountain fraction was calculated. The scenes show differing proportions of cloud versus snow. The left column in Figure 2, from the New Zealand Alps, has roughly equal fractions of cloud and snow. The middle column from the Assam Himalaya in Bhutan has many clouds. The right column from the Bernese Alps in Switzerland consists mostly of snow. The images show the error results for clouds (TPcloud, TNcloud, FPcloud, FNcloud) with a class identifier for pixels that were not cloudy. *TNcloud_is_neither* pixels are clear‐sky snow‐free pixels and *TNcloud_is_snow* pixels are clear‐sky snow‐covered pixels, with neither class mapped as cloud by the satellite. *FPcloud_is_neither* pixels are clear‐sky snow‐free pixels and *FPcloud_is_snow* pixels are clear‐sky snow‐covered pixels, with both classes mapped as cloud by the satellite. *FNcloud* are cloud pixels mapped as something else, *FNcloud*_*classed*_*snow* are a subset of this group that the satellite mapped as snow.

Table 2 shows the performance of the Landsat 8 CFMask and the MOD09GA cloud mask aggregated across all test scenes for the classes of interest, along with the ranges of values among all scenes. Generally, the Precision and Recall for the CFMask are high, whereas the Recall performance for the snow mask is lower. The MOD09GA mask does not include a snow class. In comparison to the CFMask, the MOD09GA cloud mask has a high Recall value (i.e., it sees most of the clouds) but low Precision (many false positives). The absolute value of each scene's central latitude was used to determine the proportion of variance in error statistics explained by scene location. The day of the water year, starting 1 October for Northern Hemisphere scenes and 1 April for Southern Hemisphere scenes, was used to determine the proportion of variance in error statistics explained by seasonality. *R* ^2^ values for both tests showed no significant attribute of error statistics explained by season or location.

**Table 2:** Performance of the Landsat 8 CFMask and the MOD09GA Cloud Mask

Landsat 8 CFMask					MOD09GA Cloud Mask			
Aggregated scenes					Aggregated scenes			
	Precision	Recall	*F* statistic			Precision	Recall	*F* statistic
Cloud	0.695	0.857	0.768		Cloud	0.166	0.723	0.270
Snow	0.938	0.787	0.856					
Neither	0.943	0.975	0.959		Neither	0.964	0.602	0.741

Scene‐by‐scene		Median (min, max)			Scene‐by‐scene		Median (min, max)	
	Precision	Recall	*F* statistic			Precision	Recall	*F* statistic
Cloud	0.803	0.793	0.771		Cloud	0.111	0.833	0.156
	(0.03, 0.99)	(0.03, 0.98)	(0.03, 0.96)			(0.00, 1.00)	(0.00, 1.00)	(0.00, 1.00)
Snow	0.897	0.449	0.536					
	(0.65, 1.00)	(0.00, 1.00)	(0.00, 0.98)					
Neither	0.850	0.929	0.867		Neither	0.977	0.415	0.495
	(0.00, 0.99)	(0.28, 1.00)	(0.00, 0.99)			(0.18, 1.00)	(0.00, 1.00)	(0.00, 1.00)

	Latitude versus Error (*R* ^2^)			Season versus Error (R^2^)				
Landsat 8 OLI		MOD09GA		Landsat 8 OLI		MOD09GA		
Precision	0.05	0.10	0.01			0.00		
Recall	0.03	0.01	0.22			0.01		
*F* statistic	0.01	0.01	0.03			0.00		

*Note*. Top panel shows summary statistics for CFMask and MOD09GA cloud mask performance in the test scenes for each class, by aggregate of all scenes and median, minimum, and maximum values for individual scenes. Bottom panel shows the *R* ^2^ values for individual scene errors compared to the scenes' latitudes and seasonality.

### 3.2 Reflectance Spectra of Manually Classified Pixels

**Figure 3:**
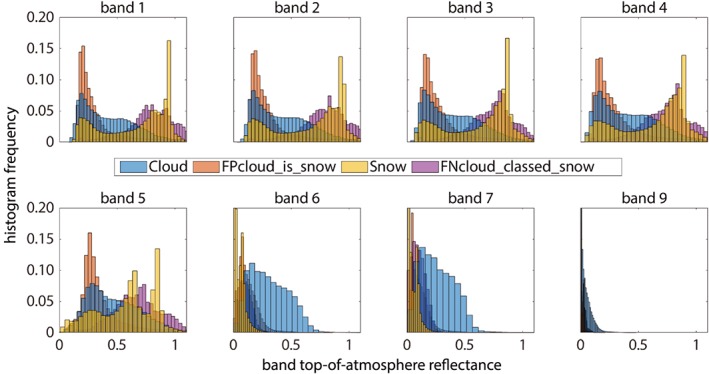
Normalized histograms of the top‐of‐atmosphere reflectance in Landsat 8 OLI bands 1–7 and 9 for manually classified snow and cloud pixels, along with histograms for misclassified pixels, combining snow classified as cloud (*FPcloud_is_snow*) and cloud classified as snow (*FNcloud_classed_snow*). The snow spectral values show a bimodal distribution with one mode in line with the skewed distribution of the cloud spectra.

Figure 3 shows histograms for top‐of‐atmosphere reflectances in Landsat OLI bands 1–7 and 9 for the manually classified cloud and snow pixels, along with the histograms for the misclassified pixels. The figure indicates that most snow and cloud spectra are easily distinguishable. Snow is brighter in visible bands and darker in SWIR bands than most cloud spectra. Yet despite the general differences, spectral characteristics of numerous pixels in each class overlap.

#### 3.2.1 Cirrus Band Reflectance From Snow at High Elevation

**Figure 4:**
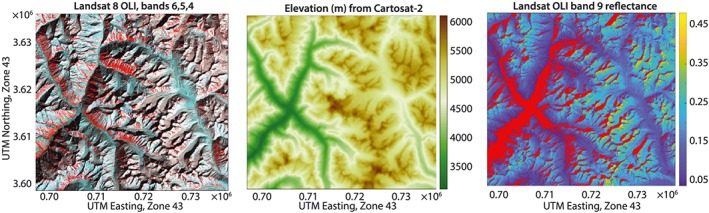
(left) False‐color rendition of Landsat 8 OLI image on 8 February 2016 in the Himachal Pradesh, India. (middle) Corresponding elevations from Cartosat‐2. (right) Values of Landsat 8 OLI band 9 top‐of‐atmosphere reflectance that exceed 0.04, with values less than 0.04 masked in red. Values above 0.04 would be classified as cloud even though there are no clouds in the frame. The use of band 9 to identify clouds will identify snow at high elevation as clouds. OLI = Operational Land Imager. UTM = Universal Transverse Mercator.

The wavelengths in the cirrus bands, Landsat OLI band 9 and MODIS band 26 (Table 1), experience absorption by atmospheric water vapor, so reflectance values in these bands are intended to map clouds high in the atmosphere. However, snow at high elevation also lies above most of the atmospheric water vapor, so the cloud masks based on putative cirrus observations often identify snow as clouds. One test for “potential cloud pixel” in the CFMask algorithm treats a top‐of‐atmosphere reflectance of 0.01 as having a 25% probability of identifying cirrus and a reflectance of 0.04 or greater with a 100% probability of cirrus (Zhu et al., 2015). These probabilities accumulate with the cloud probability scores from temperature and spectral variability. Figure 4 shows Landsat 8 OLI imagery of a 37 × 40‐km area in Himachal Pradesh, India, acquired on 8 February 2016 in support of the ISRO‐NASA AVIRIS‐NG Indian campaign (Space Applications Centre, 2017). Elevations in the area range from 3,100 to 6,100 m, and nearly all band 9 top‐of‐atmosphere reflectances exceed the CFMask's 0.04 threshold. Reflectance values from MODIS band 26 would likely be similar.

#### 3.2.2 Similar Spectra of Snow and Clouds

**Figure 5:**
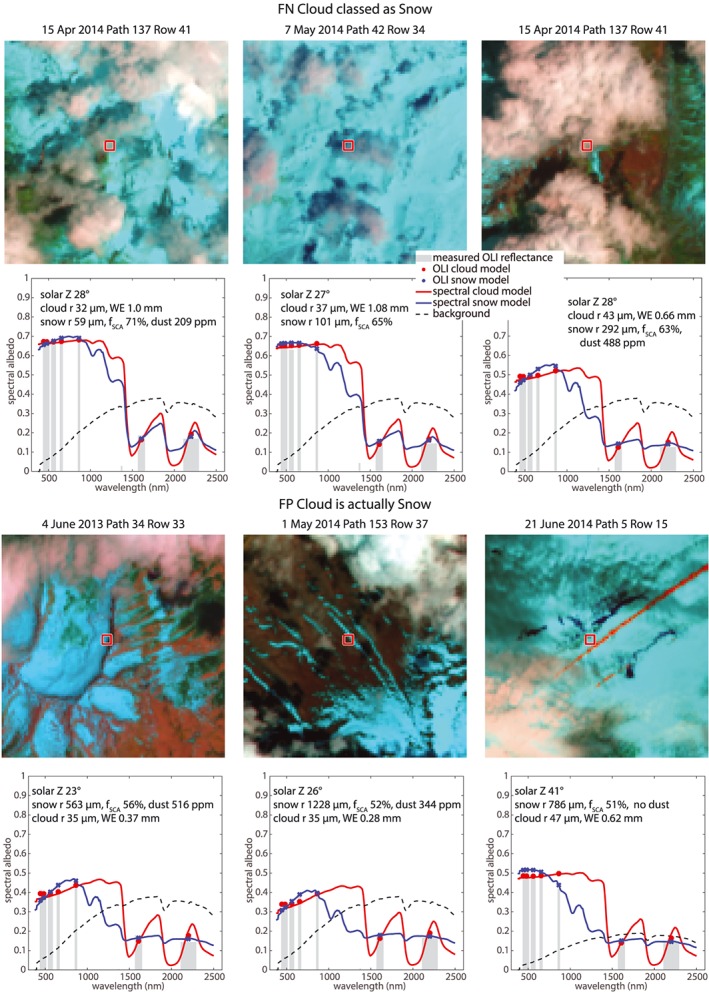
Examples of similar snow and cloud spectral signatures in the Landsat 8 OLI bands, chosen for illustration from many examples. The upper images show *FNcloud_classed_snow* (clouds were missed because they were identified as snow); the lower row of images shows *FPcloud_is_snow* (snow was misclassified as cloud). In the corresponding graphs, the gray bars show measured OLI spectra. The symbols show these spectra modeled as snow (blue) and ice clouds (red) with minuscule (10^−8^) liquid water concentration, and the text within the figures identifies the snow and cloud properties that match the spectra. *f*_*SCA*_ is the fractional snow cover of the pixel, and *WE* is the cloud water equivalent. The background reflectance is for a dark loam (Class Inceptisol, Subclass Xerumbrept). The reflectances in the Landsat bands are similar enough to obviate snow‐cloud discrimination. The blue and red lines show they would be distinguishable with continuous measurements over the wavelengths of the AVIRIS‐NG sensor, 380 to 2,500 nm at 5‐ to 6‐nm resolution. OLI = Operational Land Imager; FN = false negative; FP = false positive.

Top‐of‐atmosphere reflectances in Landsat OLI bands 6 and 7 for dark clouds are below the 99th percentile of the snow spectra in these two bands (this reflectance value is about 0.2 for both bands). Thirty‐five percent of the clouds in the spectral library meet this criterion. Figure 5 shows examples of spectra modeled as snow and cloud within the Landsat 8 OLI bands and for the wavelengths of the AVIRIS‐NG imaging spectrometer, along with the region of interest in the Landsat image where the data were acquired. We identified the snow and cloud properties by solving for values for each that minimized the Euclidean norm of the differences between measured and modeled reflectances in Landsat OLI bands 1–7. From a random sample of ~1,000 pixels with classification errors over the 26 Landsat scenes, we found that the Landsat spectral signature of 24% of the FNcloud_classed_snow pixels and 32% of the *FPcloud_snow* pixels matched plausible snow and cloud properties. All simulations considered a dark loam (Class Inceptisol, Subclass Xerumbrept) from the ECOSTRESS Library (Meerdink et al., 2019) as the surface underlying the cloud or occupying the snow‐free part of the pixel).

Although the snow and cloud spectra are distinctive if the full spectrum is considered, in the wavelength bands of the OLI they are not, and the reflectance in the cirrus band (band 9) would not clarify the spectra as snow or cloud. An imaging spectrometer, as recommended in the recent Decadal Survey for Earth Science and Applications (National Academies of Sciences, Engineering, & Medicine, 2018), could distinguish between the snow and the cloud.

**Figure 6:**
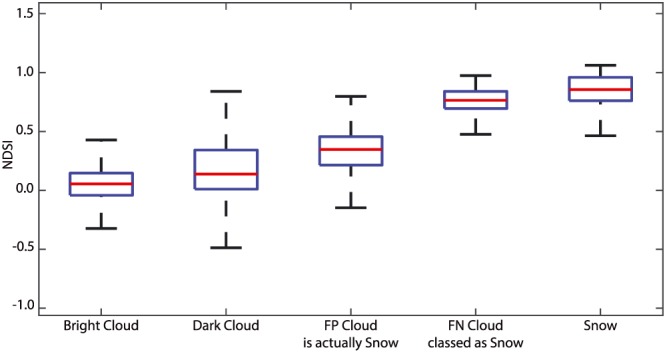
NDSI calculations for subsets of the snow and cloud spectral libraries. Outliers are not plotted, but the box‐and‐whisker plot shows overlaps among the classes. Bright clouds and snow differ significantly, whereas dark clouds and snow show enough overlap to cause misclassification. Cloud pixels classified as snow by CFMask and true snow pixels are spectrally similar. Clouds can look like snow and snow can look like clouds. NDSI = Normalized Difference Snow Index; FN = false negative; FP = false positive.

Traditional snow mapping methods often employ the NDSI (Dozier, 1989); typically the green and shortwave infrared bands are used, thus bands 3 and 6 for the Landsat 8 OLI, so *NDSI* = (*R*_3_ − *R*_6_)/(*R*_3_+*R*_6_). Figure 6 shows the range of NDSI values for various sets of pixels in the study. Snow with similar spectral characteristics to clouds has similar NDSI values, shown in the boxplots of dark cloud and false positive snow pixels, that is, the snow pixels misclassified as cloud by CFMask. Clouds with similar spectral characteristics to snow have similarly high NDSI values, shown in the similarity of boxplots of snow NDSI and the NDSI of cloud pixels that CFMask misclassifies as snow. Many of the false positive snow spectra are outliers within the context of the entire snow spectral library. Most snow has a high NDSI value between 0.5 and 1, distinguishing it from clouds, which tend to have NDSI values from −0.4 to +0.4. However, the outlier conditions that are confused with clouds comprise a source of error in scenes where the snow cover is composed of these more difficult‐to‐discriminate snow pixels.

Snow that covers an entire pixel is spectrally distinguishable from most clouds, but snow pixels that are fractional mixtures with other surface types can have spectra that are like some clouds. For fractional snow pixels, visible reflectance decreases, and SWIR reflectance increases as soil fraction increases. At the coarse spectral resolution of Landsat and MODIS, the spectra of the mixed “bright SWIR” snow pixels mimic some cloud spectra. The FPcloud_snow pixels at the borders of clouds in Figure 2 and the spectra in Figure 5 highlight this effect.

### 3.3 *k*‐Means Analyses

Table 3 shows *k*‐means performance for three runs using spectral angle to measure the similarity of snow and cloud pixels and the single run using the Euclidean norm to measure the similarity among the 35% of cloud pixels that are “dark.” The other 65% of cloud pixels are grouped as “bright clouds.” The ability to separate snow from clouds decreases as the spectra become similar. Separation, Precision, and Recall decrease when the snow is classified with the similar “dark cloud” pixels. For these most similar snow and clouds, Precision of snow and Recall of clouds improve by using a magnitude‐based measure over a spectral angle‐based measure, but performance is still worse than the other two spectral angle runs as these two sets of pixels, dark clouds and snow, are spectrally similar.

**Table 3:** Performance Statistics for the Unsupervised Classification of the Snow and “Dark Clouds,” Snow and All Clouds, and Snow and “Bright Clouds”

				Precision		Recall	
Method	Spectral libraries	Separation	Overall accuracy	Snow	Cloud	Snow	Cloud
Spectral angle	Snow/bright cloud	0.09	0.957	0.961	0.953	0.953	0.961
	Snow/cloud	0.08	0.918	0.894	0.945	0.948	0.887
	Snow/dark cloud	0.07	0.850	0.796	0.927	0.940	0.759
Euclidean norm	Snow/dark cloud		0.788	0.841	0.744	0.698	0.878

*Note*. The separation distance metric for the Euclidean norm is excluded as no comparative runs were needed.

## 4 Discussion

### 4.1 Cloud Mask Performance

Cloud masking algorithms perform worse over snow‐covered areas compared to other land surfaces because the multispectral sensor measurements can contain snow spectra indistinguishable from clouds and cloud spectra indistinguishable from snow. Significant improvement in our abilities to distinguish clouds from snow must utilize additional information beyond per pixel spectral characteristics.

Table 4 displays the results from prior cloud masking studies for comparison to the MOD09GA and CFMask performance measures from this study. CFMask performance is lower over snow‐covered mountain regions compared to previous global assessments of Landsat 8 cloud masking. The Recall and Precision of CFMask over mountains is lower compared to prior error assessments in polar regions of CFMask, the best cloud mask in these regions tested by Foga et al. (2017). The MOD09GA cloud mask has lower performance metrics compared to the CFMask results of the same scenes and to all prior studies that assessed Landsat 8 cloud mask performance. Both MODIS and Landsat scenes exhibit a spread in performance based on the local composition of each scene. Therefore, depending on the spectra of the cloud and the snow in the scene, performance can vary from excellent to unacceptable.

**Table 4:** Comparison of Error Statistics for CFMask and the MOD09GA Cloud Mask to Published Error Statistics

Cloud Mask	Precision	Recall
MOD09GA cloud mask in snow‐covered mountains (this study)	0.166	0.723
CFMask for clouds in snow‐covered mountains (this study)	0.695	0.857
CFMask for snow and ice (Foga et al., 2017, Table 7)	0.757	0.866
CFMask global assessment (Foga et al., 2017, Table 5)	0.880	0.973
Fmask global sample (Hughes & Hayes, 2014)	0.740	0.986
ACCA “best cloud mask over snow/ice” (Foga et al., 2017, Table 7)	0.706	0.993

The performance measures of CFMask to classify clear‐sky pixels without snow exceeded 95%. Low fractional snow cover leads to bright SWIR snow pixels and can be difficult to detect by a human analyst, and the false positive cloud mask pixels from CFMask that were clustered around the borders of the snow suggests that many of these pixels contained fractional snow missed by the analysts. Users should be comfortable using CFMask for identifying clear‐sky snow‐free pixels in mountain scenes unless the region of interest is the boundary around a snowpack.

The MODIS MOD09GA cloud mask has worse performance than CFMask with low Precision and moderate Recall. One plausible factor for worse performance may have been the manner in which the OLI reference mask, which we coarsened to MODIS resolution using Gaussian pyramid reduction (Burt & Adelson, 1983), was used to assign a single reference class to each MODIS pixels. We tested OLI reference mask coverage thresholds of 10% and 100% for classing MODIS pixels. This variability had moderate impact on precision (0.223 and 0.0917) and minimal impact on recall (0.699 and 0.72).

The MOD09GA algorithm misclassifies many snow pixels in each scene as cloud, and many cloud pixels are missed and can be mistaken for snow. This pervasive misclassification presents a challenge for extracting snow data from MODIS imagery. The misclassification of snow as cloud and vice versa adds bias to models of snowmelt (Andreadis & Lettenmaier, 2006) and estimates of snow water equivalent (Rittger et al., 2016) that assimilate data from MODIS. Improvement of cloud masks over snow could improve these models.

One reason for the decrease in performance compared to the Landsat 8 product is that the 500‐m cloud mask must classify more mixed pixels than the Landsat 8 30‐m cloud mask. The spatial scale of MODIS pixels creates spectral mixture problems for more pixels. Sixty percent of MODIS pixels for which the Landsat CFMask correctly classified cloud and snow had snow or cloud coverage fractions less than 100% coverage. The false positive rate of the MOD09GA cloud mask is also significantly higher than that of CFMask. Many clear‐sky, bare ground pixels were included in the MOD09GA cloud mask, so while the remaining pixels may be bare, many useable pixels of surface reflectance are mistakenly masked as cloud, a problem that was not observed in Landsat 8 imagery. Users cannot assume that MODIS pixels are either entirely cloud‐free or entirely cloud‐covered in imagery over mountains. The mixed‐pixel problem for cloud classification at 500‐m resolution is fundamentally different than at 30 m for Landsat, as mixed cloud pixels are rare at the smaller Landsat spatial scale. These larger mixed pixels in MODIS reduce the sharpness of the spectral differences between pure endmembers of snow, clouds, and other surfaces.

We did not directly analyze snow masking abilities of the MOD9GA cloud mask, because the MODIS QA bands for snow detection do not confirm snow or ice but just flag the processing path of the cloud mask algorithm for that pixel. The MODIS Collection 6 snow mask is an ancillary data product unrelated to the cloud mask. The existing snow masking for MODIS relies on the cloud mask to identify cloud pixels, and the poor results of the MOD09GA cloud mask show that any analysis of snow that removes pixels that are flagged as clouds will likely elide valid snow‐covered pixels.

The snow masking code in CFMask is based on NDSI and band 9 cirrus thresholds. Moreover, the mask is conservative towards clouds; if there is ambiguity in the algorithm logic, it classifies the pixel as cloud instead of snow. Many of the false positive snow pixels classed as cloud are outliers on the library of snow spectra, but many of these pixels are around the snowline. As the snow melts and disappears, the fractional snow‐covered area of pixels decreases, and pixels transition from the interior of the snowpack to the boundary. These are important to class as snow for modeling snow water equivalent and snowmelt runoff.

### 4.2 Spectral Library Insights

This project benefited from an extensive set of reference data that included a snow class separate from other land surfaces to test cloud mask performance. The manually classified snow and cloud masks enabled us to construct a spectral library to explore if snow and cloud can have similar spectral signatures. Our spectral library does not necessarily represent the frequency and range of possible cloud and snow spectra encountered in remote sensing imagery, but scene‐by‐scene statistics and the library of all scenes' spectra together highlight the likelihood for indiscriminate snow and cloud spectra within a scene.

The insight that spectral reflectance of 35% of the cloud pixels in the study resemble spectral reflectance of snow in the SWIR bands confirms that while most snow and cloud are spectrally distinct, the problem of spectral indiscrimination is not a fringe issue. The *k*‐means analysis compares the similarity between the two classes based on a classification technique that relies exclusively on a calculation of spectral similarity. The fractions of pixels within the spectral library that exhibit strong similarities to the general spectral characteristics of the opposite class is too large to be attributed to chance or random error. In situations where the reflectances in the SWIR bands are similar for snow and clouds, discrimination between them becomes difficult.

Spectra from pixels with large fractions of subpixel soil can resemble spectra of thin clouds, with the mixed pixels showing elevated SWIR reflectance compared to the snow within the mixture. Further anecdotal evidence lies in the band of misclassified clouds around the edges of the snowpack in some Landsat 8 images. The same pattern is observed in the MODIS data, where at 500 m many more of the snow pixels are mixtures, increasing the misclassification rate compared to Landsat. In addition to snow with elevated SWIR reflectance, clouds with low reflectance in SWIR bands are common and their NDSI values resemble those for snow. Reflectance in Landsat OLI band 9 or MODIS band 26 of snow at altitude will often have values like those of cirrus clouds.

### 4.3 Ideas for Improving Cloud Masks in Imagery From Multispectral Sensors

Resolving these types of discrimination issues requires new approaches to cloud masking that account for local viewing conditions, textural features, and persistence. This error analysis and spectral investigation helps formulate ideas for future improvements in cloud masking over snow‐covered terrain. For snow‐cloud discrimination in a single Landsat 8 or MODIS image, textural features might be used; our data set provides information to develop such approaches. Mountainous terrain itself provides spatial patterns in imagery, so one potential approach is to see whether those patterns cannot be recognized in an image because of obscuration by clouds.

The twice daily MODIS daytime acquisitions allow cloud discrimination by examining the time series, applying various filters, and interpolating to fill the gaps caused by obscuring clouds (Gafurov & Bárdossy, 2009; Helfrich et al., 2007; Tran et al., 2019). Sentinel 2a&b along with Landsat 8 provide coverage at repeat intervals of 4 days. Their coincident overpass times with MODIS Terra could offer additional validation data for MODIS and perhaps a source of training data for machine learning approaches to cloud masking with MODIS. Detection of cloud movement would be especially useful with data from geostationary satellites. The improved capabilities of the generation of GOES‐16/17 (U.S.), Himawari‐8/9 (Japan), and FengYun‐4 (China) satellites show promise for leveraging the spectral capabilities of MODIS and its successors with the high temporal frequency of geostationary satellites to mask clouds over snow.

Going forward, a reliable cloud mask is necessary to create the best snow mask. By including currently missed clouds in a cloud mask and excluding the misclassified snow, existing snow‐mapping techniques that are reliably used under clear‐sky conditions could be extended with similar performance to cloudy scenes.

### 4.4 The Case for Imaging Spectroscopy

Among the five observations in the “designated” (high priority) class recommended by the National Academies of Science, Engineering, and Medicine for future space missions is imaging spectrometry in the visible and shortwave infrared and multispectral imagery in the thermal infrared (National Academies of Sciences, Engineering, & Medicine, 2018). As Figures 4, 5, 6 show, existing multispectral sensors sometimes have similar spectral signatures for snow and clouds. Measurements of spectral reflectance across the solar spectrum from 400 to 2,500 nm at 5‐ to 10‐nm resolution would enable unambiguous discrimination with reliable estimates of soil or vegetative background reflectance, the sizes of the scattering ice crystals or water droplets, fractional snow cover, thickness of clouds, and degradation of snow albedo by dust or soot.

## 5 Conclusion

Satellite measurements provide our best resource for furthering our understanding of the cryosphere. Cloud masking algorithms over snow‐covered terrain perform poorly compared to over other land surfaces, and existing cloud masking performance depends on local cloud and snow types and cannot deliver consistently accurate results. In multispectral images of the visible and infrared spectra, many clouds and snow pixels are unambiguously separable, mainly for thick clouds and snow that fully covers a pixel. However, variations in snow and cloud properties can cause overlap in their spectral signatures within a scene. While multispectral differences alone can identify many pixels correctly as cloud or snow, others remain ambiguous. Landsat 8 OLI data confirm the possibility of cloud spectra like snow or snow spectra like cloud as a plausible source of algorithm misclassifications. Availability of imagery from a future spaceborne imaging spectrometer would provide validation data to evaluate new multispectral methods for snow‐cloud discrimination.

## Data

This paper does not exhaust the ways in which the data we have assembled could be used to understand snow‐cloud discrimination. Although we plan to explore new methods using these data, all data that we created are available (at Stillinger & Collar, 2019) to other researchers interested in the same problem. Table S1 lists all 26 Landsat 8 OLI and corresponding MODIS images analyzed in this study. The data we provide with unrestricted access include the geoTIFFs where we have manually identified snow and clouds in 13 images. The collection from the U.S. Geological Survey (2016) includes full‐resolution images from their manual classification. We also provide the spectral libraries for clouds, snow, false positives, and false negatives in the manually classified imagery. MATLAB code for the snow albedo model (based on Wiscombe & Warren, 1980) is available on GitHub at the website (https://github.com/edwardbair/SCAGD/). The SMARTS model for atmospheric radiation is available from the National Renewable Energy Laboratory (https://www.nrel.gov/grid/solar-resource/smarts.html). The ECOSTRESS spectral library of natural and human‐made materials is available from JPL (https://speclib.jpl.nasa.gov/).

## Supporting information



Supporting Information S1
