# Supplementary material for: Cloud Masking for Landsat 8 and MODIS Terra Over Snow‐Covered Terrain: Error Analysis and Spectral Similarity Between Snow and Cloud
Source: Water Resour Res. 2019 Jul 29;55(7):6169–84. doi: 10.1029/2019WR024932 (PMC6988483; doi:10.1029/2019WR024932)
Supplement: Supplementary file 1 — Supporting Information S1 [file WRCR-55-6169-s001.docx]

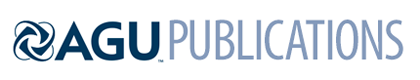


*Water Resources Research*

Supporting Information for

**Cloud Masking for Landsat 8 and MODIS Terra over Snow-Covered Terrain: Error Analysis and Spectral Similarity between Snow and Cloud**

Timbo Stillinger^1^, Dar A. Roberts^2^, Natalie M. Collar^1†^, and Jeff Dozier^1^

^1^Bren School of Environmental Science & Management, University of California, Santa Barbara, California, USA. ^2^Department of Geography, University of California, Santa Barbara, California, USA.

^†^Current address Wright Water Engineers Inc., Denver, Colorado, USA, and Department of Civil and Environmental Engineering, Colorado School of Mines, Golden, Colorado, USA.

**Contents of this file**

Table S1

Table S1. Statistics for Precision, Recall, and the F-statistic for CFMask for all Landsat 8 OLI images and the MODIS cloud mask for all MOD09GA images analyzed.

| Date | Sensor/ Product | Scene Coordinates | Mountain Cover | Geographic Coordinates | | *Precision* | *Recall* | *F statistic* |
| --- | --- | --- | --- | --- | --- | --- | --- | --- |
| 13-Jan-2014 | Landsat 8 OLI | WRS P 76 R 91 | 67% | -44.5 | 168.3 | 0.765 | 0.892 | 0.824 |
|  | MOD09GA | MODIS h29v13 & h30v13 |  |  |  | 0.246 | 0.845 | 0.381 |
| 15-Apr-2014 | Landsat 8 OLI | WRS P 137 R 41 | 90% | 27.8 | 90.8 | 0.856 | 0.965 | 0.907 |
|  | MOD09GA | MODIS h26v06 |  |  |  | 0.553 | 0.997 | 0.711 |
| 5-Jun-2013 | Landsat 8 OLI | WRS P 195 R 28 | 89% | 46.4 | 7.8 | 0.766 | 0.939 | 0.844 |
|  | MOD09GA | MODIS h18v04 |  |  |  | 0.101 | 0.907 | 0.182 |
| 1-Apr-2014 | Landsat 8 OLI | WRS P 167 R 51 | 60% | 47.8 | -113.9 | 0.992 | 0.801 | 0.887 |
|  | MOD09GA | MODIS h22v07 |  |  |  |  | 0.000 | 0.000 |
|  |  |  |  |  |  |  |  |  |
| 1-May-2014 | Landsat 8 OLI | WRS P 153 R 37 | 79% | 49.0 | -113.6 | 0.934 | 0.896 | 0.915 |
|  | MOD09GA | MODIS h23v05 |  |  |  | 0.018 | 1.000 | 0.036 |
|  |  |  |  |  |  |  |  |  |
|  |  |  |  |  |  |  |  |  |
| 4-Dec-2014 | Landsat 8 OLI | WRS P 152 R 35 | 100% | 39.3 | -107.7 | 0.999 | 0.726 | 0.841 |
|  | MOD09GA | MODIS h23v05 |  |  |  | 0.084 | 1.000 | 0.156 |
|  |  |  |  |  |  |  |  |  |
| 4-Jun-2013 | Landsat 8 OLI | WRS P 35 R 33 | 95% | 51.8 | -72.9 | 0.960 | 0.810 | 0.879 |
|  | MOD09GA | MODIS h09v05 |  |  |  | 0.003 | 0.100 | 0.005 |
|  |  |  |  |  |  |  |  |  |
| 4-Jun-2013 | Landsat 8 OLI | WRS P 35 R 34 | 76% | 49.7 | -122.2 | 0.969 | 0.861 | 0.912 |
|  | MOD09GA | MODIS h09v05 |  |  |  | 0.000 | 0.000 | 0.000 |
|  |  |  |  |  |  |  |  |  |
| 5-Apr-2014 | Landsat 8 OLI | WRS P 42 R 34 | 89% | 36.7 | 76.3 | 0.998 | 0.905 | 0.949 |
|  | MOD09GA | MODIS h08v05 |  |  |  | 0.000 | 0.000 | 0.000 |
|  |  |  |  |  |  |  |  |  |
| 6-Sep-2014 | Landsat 8 OLI | WRS P 217 R 15 | 35% | 27.7 | 84.8 | 0.034 | 0.030 | 0.032 |
|  | MOD09GA | MODIS h17v02 |  |  |  | 0.228 | 0.676 | 0.341 |
|  |  |  |  |  |  |  |  |  |
| 7-May-2014 | Landsat 8 OLI | WRS P 42 R 33 | 87% | 27.8 | 90.8 | 0.747 | 0.906 | 0.819 |
|  | MOD09GA | MODIS h08v05 |  |  |  | 0.227 | 0.833 | 0.357 |
|  |  |  |  |  |  |  |  |  |
| 7-May-2014 | Landsat 8 OLI | WRS P 42 R 34 | 89% | 36.7 | 76.3 | 0.802 | 0.859 | 0.829 |
|  | MOD09GA | MODIS h08v05 |  |  |  | 0.202 | 1.000 | 0.337 |
|  |  |  |  |  |  |  |  |  |
| 10-Dec-2013 | Landsat 8 OLI | WRS P 231 R 94 | 80% | 30.4 | 78.2 | 0.778 | 0.894 | 0.832 |
|  | MOD09GA | MODIS h13v13 |  |  |  | 0.165 | 0.847 | 0.276 |
|  |  |  |  |  |  |  |  |  |
| 11-Apr-2013 | Landsat 8 OLI | WRS P 146 R 39 | 75% | 46.5 | 7.8 | 0.922 | 0.896 | 0.909 |
|  | MOD09GA | MODIS h24v05 & h24v06 |  |  |  | 0.833 | 1.000 | 0.909 |
|  |  |  |  |  |  |  |  |  |
|  |  |  |  |  |  |  |  |  |
| 14-Aug-2014 | Landsat 8 OLI | WRS P 47 R 25 | 98% | 33.8 | 74.3 | 0.792 | 0.953 | 0.865 |
|  | MOD09GA | MODIS h10v04 |  |  |  | 0.564 | 0.872 | 0.685 |
|  |  |  |  |  |  |  |  |  |
| 14-Jul-2013 | Landsat 8 OLI | WRS P 148 R 35 | 100% | 64.8 | -17.0 | 0.865 | 0.780 | 0.820 |
|  | MOD09GA | MODIS h24v05 |  |  |  | 0.111 | 0.038 | 0.057 |
|  |  |  |  |  |  |  |  |  |
| 14-May-2013 | Landsat 8 OLI | WRS P 153 R 35 | 94% | 37.5 | -108.6 | 0.984 | 0.595 | 0.742 |
|  | MOD09GA | MODIS h23v05 |  |  |  |  | 0.000 | 0.000 |
|  |  |  |  |  |  |  |  |  |
| 21-Jun-2014 | Landsat 8 OLI | WRS P 5 R 15 | 0% | -70.4 | -65.8 | 0.707 | 0.136 | 0.228 |
|  | MOD09GA | MODIS h15v02 |  |  |  | 0.013 | 0.059 | 0.021 |
|  |  |  |  |  |  |  |  |  |
| 21-Mar-2014 | Landsat 8 OLI | p170r025 | 0% | 38.4 | -119.4 | 0.685 | 0.670 | 0.677 |
|  | MOD09GA | MODIS h20v03 & h21v03 |  |  |  | 0.000 | 0.000 | 0.000 |
|  |  |  |  |  |  |  |  |  |
|  |  |  |  |  |  |  |  |  |
| 26-Dec-2013 | Landsat 8 OLI | WRS P 215 R 110 | -* | 37.6 | -118.3 | 0.056 | 0.860 | 0.106 |
|  | MOD09GA | MODIS h15v16 |  |  |  |  |  |  |
|  |  |  |  |  |  |  |  |  |
|  |  |  |  |  |  |  |  |  |
| 26-May-2014 | Landsat 8 OLI | WRS P 15 R 24 | 8% | 63.7 | -49.3 | 0.706 | 0.906 | 0.794 |
|  | MOD09GA | MODIS h13v03 |  |  |  | 0.240 | 0.826 | 0.372 |
|  |  |  |  |  |  |  |  |  |
| 28-Dec-2013 | Landsat 8 OLI | WRS P 149 R 37 | 67% | -49.1 | -73.0 | 0.807 | 0.852 | 0.829 |
|  | MOD09GA | MODIS h24v05 |  |  |  | 0.317 | 0.626 | 0.421 |
|  |  |  |  |  |  |  |  |  |
| 29-May-2013 | Landsat 8 OLI | WRS P 41 R 27 | 96% | -44.5 | 168.3 | 0.991 | 0.866 | 0.925 |
|  | MOD09GA | MODIS h10v04 |  |  |  | 0.066 | 1.000 | 0.123 |
|  |  |  |  |  |  |  |  |  |
| 29-May-2013 | Landsat 8 OLI | WRS P 146 R 39 | 75% | 46.5 | 7.8 | 0.883 | 0.894 | 0.888 |
|  | MOD09GA | MODIS h24v05 & h24v06 |  |  |  | 1.000 | 1.000 | 1.000 |
|  |  |  |  |  |  |  |  |  |
|  |  |  |  |  |  |  |  |  |
| 30-Apr-2015 | Landsat 8 OLI | WRS P 141 R 41 | 65% | 50.5 | 47.5 | 0.940 | 0.984 | 0.961 |
|  | MOD09GA | MODIS h25v06 |  |  |  | 0.018 | 1.000 | 0.036 |
|  |  |  |  |  |  |  |  |  |
| 30-Jun-2013 | Landsat 8 OLI | P 41 R 26 | 59% | -44.5 | 168.3 | 0.940 | 0.737 | 0.826 |
|  | MOD09GA | MODIS h10v04 |  |  |  | 0.000 | 0.000 | 0.000 |
|  |  |  |  |  |  |  |  |  |
